# Surgical and Biological Treatment with a Platelet-Rich Fibrin Matrix for Patellar Tendinopathy: Clinical Outcomes and Return to Sport at 2-Year Follow-Up

**DOI:** 10.3390/jpm14060567

**Published:** 2024-05-25

**Authors:** Venanzio Iacono, Luca Padovani, Fjorela Qordja, Luca De Berardinis, Daniele Screpis, Antonio Pompilio Gigante, Claudio Zorzi

**Affiliations:** 1 Department of Orthopaedics, IRCCS Ospedale Sacro Cuore Don Calabria, 37024 Negrar, Italy; venanzio.iacono@sacrocuore.it (V.I.); luca.padovani@sacrocuore.it (L.P.); daniele.screpis@sacrocuore.it (D.S.); claudio.zorzi@sacrocuore.it (C.Z.); 2 Clinical Orthopaedics, Department of Clinical and Molecular Sciences, Università Politecnica delle Marche, 60126 Ancona, Italy; luca8191deberardinis@gmail.com (L.D.B.); a.p.gigante@staff.univpm.it (A.P.G.)

**Keywords:** patellar tendinopathy, jumper’s knee, platelet-rich fibrin matrix, PRFM, orthobiology, return to sport, VISA-P, Blazina score

## Abstract

Background: Patellar tendinopathy (PT) involves anterior knee pain and functional. Platelet-rich fibrin matrix (PRFM) is a promising biological therapy for tendinopathies. We examined a cohort of PT patients treated with tendon debridement and autologous PRFM at the 24-month follow-up to assess whether the combined treatment facilitated return to sports and yielded satisfactory clinical and functional scores. Methods: Baseline and 24-month visual analogue scale (VAS), Victorian Institute of Sport Assessment Scale for Patellar Tendinopathy (VISA-P), Tegner Activity Scale (TAS), and Blazina scores were compared to evaluate treatment effectiveness. The Friedman test was used to compare repeated observations of VAS, VISA-P, TAS, and Blazina Score values. Return to sport rate, Tampa Scale of Kinesiophobia (TKS) score and patient satisfaction were collected at 24 months. Results: The postoperative clinical scores demonstrated significant improvement compared with their preoperative values (all *p* < 0.001). Specifically, the VISA-P score was 80.32 (±20.58), 92.10% of patients had resumed sports activities and patient satisfaction was 9.21 (±1.21) at 24 months. Conclusions: Surgical debridement and autologous PRFM application in patients with chronic PT resulted in a higher rate of return to sports when compared to solely surgical treatment, significantly improved clinical outcomes and excellent patient satisfaction at 24 months.

## 1. Introduction

Patellar tendinopathy (PT) is a common cause of anterior knee pain and functional impairment arising from cyclic mechanical stress on the patellar tendon. PT often develops as a reaction to excessive usage, hence its lay name of “jumper’s knee”, and is characterized by a complicated, multi-faceted pathology [1].

PT is three times as frequent (5.8%) as Achilles tendinopathy (1.8%) among adolescent athletes and has been found to exceed 28% in a group of individuals aged 50–79 years with no history of knee injury [2].

Intrinsic risk factors for PT include male gender, higher body weight, body mass index (BMI) and waist-to-hip ratio, leg length discrepancy, foot arch height, vertical jump ability, and flexibility and strength of the hamstring and quadriceps. A high patella and an enlarged infrapatellar fat pad also increase the risk in males.

The disorder has been described as a continuum [3]. An acute overload or direct impact triggers the reactive stage, characterized by a non-inflammatory response with an increase in cells and matrix causing temporary swelling and pain. Adequate load reduction or sufficient rest can reverse the situation. Otherwise, in the next stage, which is tendon disrepair, the healing mechanism involves an increase in cell activity and protein production that result in collagen separation and matrix disorganization. This stage, though concerning, is still reversible with proper load management. In the final phase, the degenerative stage, cell apoptosis, extensive changes in the matrix and the vascular structure offer limited reversibility. This stage is associated with recurrent pain and, due to the extensive degenerative changes, a high risk of tendon rupture.

The PT diagnostic workup begins with history taking, initially focusing on patient age, the location of the pain, which is usually the inferior pole of the patella [4], its onset pattern, and the type of sport activity in which the patient engages. On physical examination, tenderness over the patellar tendon adds another important detail. There are three key tests to assess PT. The passive flexion-extension sign [5] involves checking for tenderness in the patellar tendon in different conditions of knee flexion and extension, a positive result being the decrease in tenderness when the knee is flexed. The standing active quadriceps sign [6] tests for changes in tenderness as the patient, standing upright, flexes the knee. Finally, the single leg decline squat test [7], especially effective when combined with pain mapping, requires the patient to squat on a tilted board until pain arises, to evaluate its onset. The entire lower limb is assessed for any biomechanical issues involving the hip, knee, foot, and ankle. A magnetic resonance imaging (MRI) scan can help to confirm PT, the expected diagnostic indicators including tendon hypertrophy and hyperintense fluid signal on T2-weighted sequences, whereas attenuation phenomena may occur in the context of significant tears [8]. Finally, a tendon thickness exceeding 7 mm has been suggested to be clinically significant in both ultrasound and MRI scans [9].

It is imperative to adopt an optimal management strategy as soon as PT manifests, to avert chronicization, as chronic pain significantly affects quality of life [10]. Although tendons possess an intrinsic natural healing capacity, repair often involves the formation of areas of tendinosis, which compromise their mechanical integrity and elasticity and impair their performance. Furthermore, the fibrosis makes tendons more vulnerable to subsequent injuries, hampering the achievement of full functional recovery. The high complexity and incidence of PT underscore the need for innovative therapeutic strategies that support effective tendon healing while minimizing the tendinosis process.

The starting point for treatment includes conservative treatment; of the various conservative options, exercise therapy is the most effective for PT. Besides traditional eccentric exercise, promising progressive tendon-loading exercise therapy programs consisting of daily isometric, isotonic (dynamic), energy storage (explosive), and sport-specific exercises are now emerging. Isometric and isotonic exercise, patellar straps, sports tape, and kinesiotaping have short-term effects, whereas progressive tendon-loading exercise, dry needling, platelet-rich plasma (PRP), and ESW therapy provide long-term effects [11].

Although the majority of individuals with PT respond well to conservative treatment, surgery is needed for the approximately 10% who do not show marked improvement [12].

Tissue engineering and regenerative medicine approaches seek to avoid surgical interventions. They use sophisticated scaffolding systems that aim at rapid and effective healing of tissue injuries in any area of the body. The integration of innovative materials and biological components, ranging from bone marrow aspirate concentrate [13] and microfragmented adipose tissue [14,15] to advanced biomaterials, especially platelet-rich fibrin matrices (PRFMs), has sparked considerable interest [16].

Biological augmentation with PRP and its derivatives has successfully been used to manage musculoskeletal disorders since the early 2000s. PRP, obtained by processing the patient’s blood, contains growth factors such as platelet-derived growth factor (PDGF), transforming growth factor-beta (TGF-β), fibroblast growth factor (FGF), insulin-like growth factor (IGF), vascular endothelial growth factor (VEGF), and endothelial cell growth factor (ECGF). Such factors significantly enhance cell proliferation and migration, angiogenesis, and extracellular matrix production in a variety of cell types both in vivo and in vitro, enhancing tissue repair and regeneration processes [17,18].

There are four classes of PRP products, in relation to their fibrin architecture and leukocyte content: pure PRP, leukocyte and PRP (L-PRP), pure platelet-rich fibrin (PRF), and leukocyte and PRF (L-PRF). The latter has the ability to release growth factors gradually over a period of 7 days; moreover, the growth factors, platelets, and leukocytes contained in the complex fibrin matrix appear to provide a synergistic action that potentiates tissue repair and regeneration [19].

Autologous PRFMs may be a promising method to deliver locally increased concentrations of platelet-derived growth factors and other bioactive molecules over a prolonged period of time, combining the benefits of PRP and PRF. PRFMs involve processing steps that allow obtaining a spongy, gel-like substance or fibrin matrix, which ensures sustained release of growth factors and offers a supportive structure for cells, enhancing tissue healing. The surgical treatment of PT combined with autologous PRFM systems could offer novel avenues for improved healing and functional restoration. PRFMs have already been applied to enhance the healing of muscle [20], the Achilles tendon [21,22], the rotator cuff tendon [23], and meniscal injuries [19] with variable results.

The RegenKit Extracell Membrane (Regen Lab SA, Le Mont-sur-Lausanne, Switzerland) is a fully autologous platelet biological matrix that boasts a high concentration of viable platelets. It is obtained from a minimal volume of the patient’s blood, which undergoes centrifugation to produce a dense, suturable PRFM for direct application to the lesion, be it a soft tissue or a bone lesion.

This clinical study assesses pain, functional outcomes, return to sport and level of activity at 2-year follow-up in a cohort of individuals with PT treated by surgical scarification and PRFM augmentation.

## 2. Materials and Methods

### 2.1. Study Design

This is the first case series involving patients diagnosed with chronic PT who were managed with surgical scarification and biological augmentation using a PRFM system. The study was conducted at IRCCS Ospedale Sacro Cuore “Don Calabria” (Negrar, Italy) from 2015, when we began using the RegenKit, to 2022. The study received approval from the Ethics Board of Verona and Rovigo (protocol no. 61386–19/09/2018). The clinical research was in accordance with the Declaration of Helsinki in 2013. All patients provided their signed informed consent.

### 2.2. Patient Selection

The inclusion criterion was chronic anterior knee pain due to chronic PT according to Kaux et al. [24] (i.e., symptom duration > 3 months), not responsive to conservative treatment.

Exclusion criteria were absence of a preoperative MRI scan supporting the anamnestic-clinical diagnosis of PT and/or of a 6-month follow-up scan obtained with the same field strength as our institute’s; bilateral PT (diagnosis or treatment); a history of surgical interventions to the knee, and a follow-up of less than 2 years. We excluded all major differential diagnoses for anterior knee pain and PT, i.e., patellofemoral syndrome, hoffitis (fat pad syndrome), traction apophysitis, be it Osgood–Schlatter disease (affecting the anterior tibial tuberosity) or Sinding–Larsen–Johansson syndrome (affecting the lower pole of the patella), chondral or meniscal lesions, quadriceps tendinopathy, synovial plica, and referred pain. Further exclusion criteria were the manufacturer’s absolute contraindications for the use of the RegenKit Extracell Membrane, i.e., platelet dysfunction syndrome, critical thrombocytopenia, hemodynamic instability, severe metabolic or systemic disorders, septicemia, acute/local infection at the site of the procedure, and the patient’s unwillingness to accept risks.

**Figure 1:**
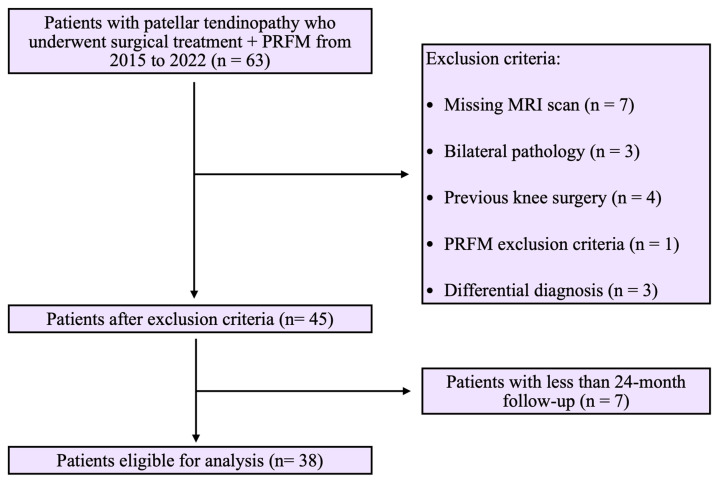
Patient selection flowchart.

The patient selection flow-chart is reported in Figure 1.

### 2.3. PRFM Preparation

Before taking the patient to the operating room, PRFM is prepared according to the manufacturer’s guidelines. First, approximately 10 mL of the patient’s blood, drawn via venous puncture, is centrifuged at room temperature for 9 min at 1500× *g*, to separate the red blood cells and the granulocytes, which remain trapped under the gel, from the platelets and the blood mononuclear cells, which settle on the surface. The next step involves homogenizing the mixture by gently flipping the tubes several times, to induce resuspension of the cellular sediment in the plasma supernatant. Approximately 5.5 mL of the mixture, extracted into a syringe from each tube, is added with 0.5–1 mL of 10% *w*/*v* calcium gluconate solution per tube, to promote fibrin polymerization. A second centrifugation is then performed at room temperature at 1500× *g* for more than 20 min. A 10-min centrifugation yields a dense coagulum, whereas the next 10 min cause the coagulum to break down, resulting in the formation of a fibrin membrane that is carefully collected using a sterile forceps.

### 2.4. Surgical Technique

**Figure 2:**
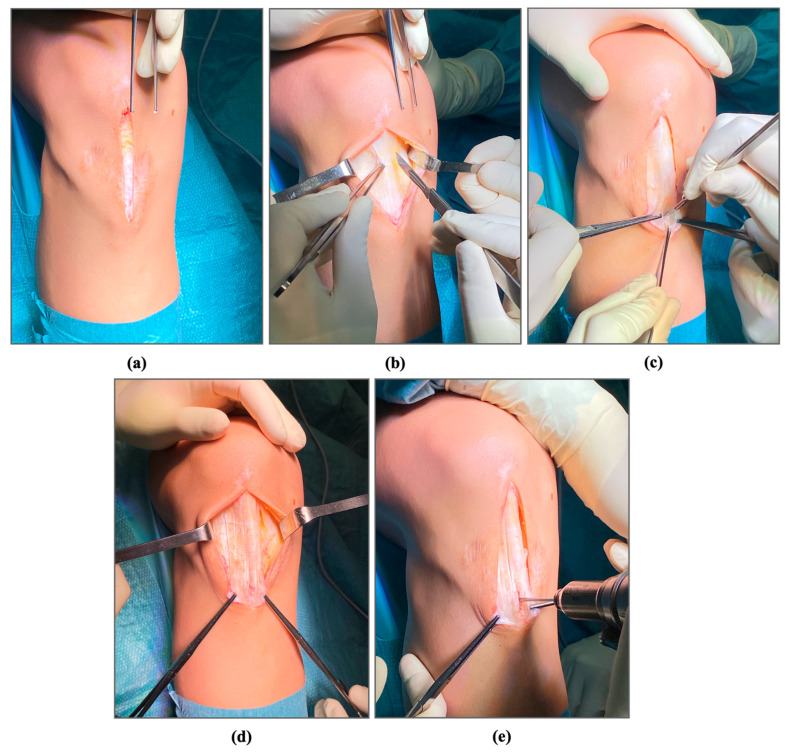
Relevant steps during the surgical debridement: (**a**) Surgical incision; (**b**) identification of the peritenon; (**c**) excision of the areas of tendinosis; (**d**) fenestrations; (**e**) microfractures.

The patient, under spinal anesthesia, is placed in supine position on the operating table with sterile draping around the leg and a well-padded thigh tourniquet placed high and inflated to 300 mm Hg. Infection prophylaxis is with the standard dose of perioperative cefazolin. With the knee flexed at approximately 90°, we perform a midline longitudinal incision from the inferior pole of the patella distally for 3–4 cm (Figure 2a). The prepatellar bursa and any other fibrous tissue overlying the proximal tendon is sharply excised.

We then open the peritenon and perform a careful medial and lateral dissection. The peritenon is important for the healing process and should be protected and sutured at the end of the procedure (Figure 2b) [25]. The medial and lateral borders of the patellar tendon are palpated, and a midline incision is made at the level of the thickened, tendinosis area, which is sharply excised (Figure 2c). During the excision, it is important to keep the knee at 90° of flexion, to ensure adequate tension. We then perform fenestrations above and below the areas that have been excised, to foster the healing response (Figure 2d). In patients with proximal or distal tendinopathy, microfractures are made with a surgical drill on the inferior pole of the patella or the tibial tubercle, through the area above or below the tendon incision, to stimulate new blood supply (Figure 2e).

**Figure 3:**
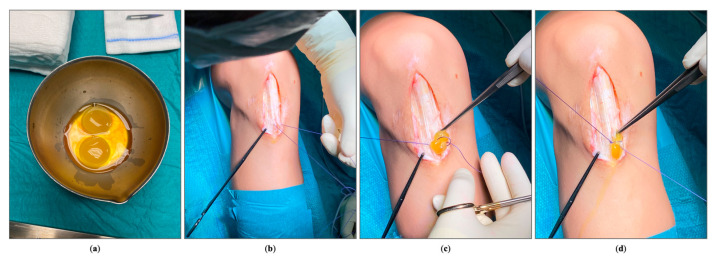
Suture of the PRFM on the residual tendon: (**a**) PRFM; (**b**) suture preparation; (**c**) PRFM suture on the abraded intra-tendinous region; (**d**) tight of the knot.

Then, the autologous PRFM membrane is applied on the surgical site in two layers. First on the freshly abraded intra-tendinous region (Figure 3), where the membrane is sutured with absorbable No. 2 Vicryl sutures (Ethicon, Somerville, NJ, USA) to the area of the tendon where the tendinosis tissue has been excised.

**Figure 4:**
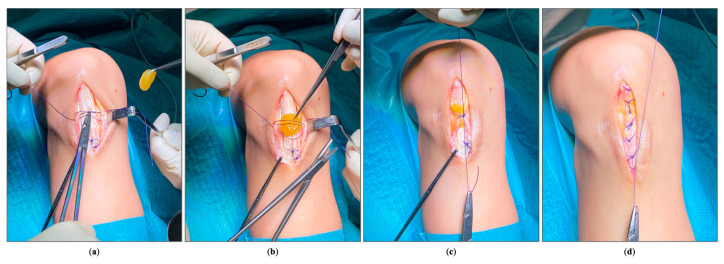
Suturing the PRFM to the peritenon: (**a**) knot preparation; (**b**) PRFM application; (**c**) PRFM suture at the level of the peritenon; (**d**) complete suture of the peritenon.

Secondly, at the level of the peritenon (Figure 4), with an identical suture, to provide biological support to the area around the surgical wound.

Discharge is on the following day if no complications have arisen.

### 2.5. Rehabilitation Protocol

Physical therapy, wearing a knee brace, begins the morning after the operation with passive isometric quadriceps exercises as soon as the patient feels comfortable. The aims in this early phase are to activate the quadriceps, manage the edema, and control pain. The range of motion allowed in the first week is 0–30° of flexion, with gradual increases of 30° each week. Weight bearing is not allowed until the second week and is then gradually increased by 25% of body weight each week [26]. Patients receive low molecular weight heparin as prophylaxis against deep vein thrombosis for at least two weeks, or until weight bearing begins. Two weeks after the procedure, we assess the patients to remove the skin sutures. At this time, we recommend beginning active mobilization. Four to five weeks after the procedure, those patients who have regained full active and passive motion can begin concentric exercises, and subsequently proprioceptive and functional exercises and strength training. Eccentric exercises and running commence gradually after six weeks. On the eighth week, we reassessed patients and, if they have achieved adequate strength and balance, authorize their return to sports. If the patient is symptom-free, the final follow-up visit involves examination of the six-month MRI scan. To prevent symptom recurrence, it is crucial to monitor and correct any imbalance between the extensor and flexor muscles, stretch any shortened muscle groups, adjust the physical workload, and improve proprioception and sport-specific movements before full resumption of sports activities [26].

### 2.6. Outcomes

Two years after the procedure, we asked patients to rate their operation using five validated self-administered questionnaires: the visual analog scale (VAS), the Victorian Institute of Sports Assessment for Patellar Tendinopathy questionnaire (VISA-P), the Blazina Score as modified in 2002 by Ferretti et al. [27], The Tegner Activity Scale (TAS) and the Tampa Scale of Kinesiofobia (TSK). The first four questionnaires asked questions regarding the period before the operation and the end of the 2-year follow-up, whereas the TKS asked about patients’ fear of reinjury at 2 years.

The VAS is a sensitive, subjective tool [28]. It can measure the patient’s pain experience at the beginning, during rehabilitation and at the final stages, offering precise indications about its intensity and variations [29].

The VISA-P is an 8-item questionnaire designed to assess PT symptom severity and its impact on physical function [30]. Scores range from 0 to 100, the lower scores indicating greater disability.

The Blazina Score as modified by Ferretti et al. provides a thorough qualitative description of the clinical progression of a patellar tendon injury. The changes to the original classification are as follows:

- Stage 0: no pain;
- Stage 1: pain only after intense sports activity with no functional impairment;
- Stage 2: moderate pain during sports activity with no restriction on sports performance;
- Stage 3: pain with slight restriction on performance;
- Stage 4: pain with severe restriction of sports performance;
- Stage 5: pain during daily activity and inability to participate in sport at any level.

Participants’ fear of movement and of incurring a new injury, or kinesiophobia, was captured using the 2-factor 13-item TSK, where the fear of movement domain is separated from other conceptual domains such as pain, function, and mood alterations [30]. Scores range from 13 to 52 points, with higher scores indicating a greater fear of movement and re-injury. Pilot studies have found that the majority of patients with PT have clinically significant levels of kinesiophobia. The TAS is a subjective satisfaction score where patients rate their perception of the general function of the operated knee. The activity level is scored from 0 to 10, where 10 is perfect and 0 corresponds to disability. The TAS arbitrarily groups sports activities into different levels without considering their frequency [31].

Any complications or recurrences were carefully reported in the database.

### 2.7. Statistical Analysis

We used Microsoft Excel (version 16.75.2, Redmond, WA, USA) in conjunction with the XLSTAT resource pack (XLSTAT-Premium, Addinsoft Inc., New York, NY, USA) for all analyses. We employed the Shapiro–Wilk test to evaluate whether the data exhibited a non-parametric distribution. We computed mean values for all continuous data and used percentage frequencies for qualitative variables. We compared baseline and postoperative clinical scores using the non-parametric Friedman test—a method developed to analyze repeated measures—to assess differences in the VAS, VISA-P, TAS, and Blazina scores between baseline and 24 months, whereas return to sports, TKS, and patient satisfaction were assessed only at 24 months. A *p*-value < 0.05 was statistically significant.

### 2.8. Study Endpoints

The endpoints of the study, at the 2-year follow-up, were (*i*) to assess whether the treatment had provided pain relief; (*ii*), to evaluate functional and psychological outcomes; (*iii*), to analyze return to sport rates.

## 3. Results

### 3.1. Demographic Characteristics

The demographic characteristics of our cohort of patients are reported in Table 1.

### 3.2. Clinical and Functional Outcomes

As regards the study’s primary endpoint, i.e., pain relief, the combination of surgical treatment and application of a PRFM system resulted in a significant (*p* < 0.001) reduction of the mean pain score from 8.58 (standard deviation, SD, 0.89) to 1.68 (SD 2.08).

The second endpoint, namely the functional scores, also showed a significant improvement compared with the preoperative period, as shown in Table 2.

### 3.3. Return to Sport

As concerns the third endpoint, Table 3 shows that 92.10% of patients returned to sports. In this regard, it is interesting to note that the three patients who did not return were those with the highest TKS scores (37 points). However, not all patients were able to return to the same level of sport engagement as before PT. In fact, the TAS showed a reduction from the baseline value of 7.79 (SD 1.53) to 7.08 (SD 1.94).

Comparison of the MRI scans taken before surgery and at 6 months demonstrated the disappearance of the characteristic signal in the posterior region of the proximal patellar tendon and the inferior pole of the patella, with the restoration of normal tendon thickness.

No complications or severe adverse events related to the surgical procedure or to PRFM application were observed during treatment or follow-up.

## 4. Discussion

To the best of the authors’ knowledge, this is the first study describing the clinical and functional outcomes and the rate of return to sports of a cohort of patients with chronic PT who were managed by a combination of surgical treatment and the application of a PRFM membrane. The combination is not new in itself, as it has successfully enhanced the surgical outcome in other types of lesion. Rather, the novelty lies in the application of the orthobiologic treatment to tendons where the tendinosis tissue has been excised.

We achieved excellent results for all endpoints, without complications or adverse events related either to tendon scarification or to the application of the PRFM membrane. In the absence of similar studies, we compared the results of our patients with those of current treatment options for PT.

As regards pain control, Dan et al. [32] found a mean VAS score of 1.3 (SD 0.8) 12 months after surgical treatment. This score is in line with our results at 24 months. Where the functional scores are concerned, the mean VISA-P scores after surgical treatment ranged from 58.9 (SD 22.7) at six months to 72.9 (SD 11.7) at 12 months [32], and then to 88 (SD 7.1 ± 2.5) at 24 months [33]. Our treatment strategy provided excellent functional results as reflected by the VISA-P score, with mean values of 80.32 (SD 20.58). Considering the short (2-year) follow-up, it is impossible to make predictions about longer-term trends. At 22-month follow-up, Cucurulo et al. [34] found that of the patients who underwent surgery for PT, 6% had Blazina stage 3, 6% had stage 2, 18% had stage 1, and 70% were completely asymptomatic. Again, our data are consistent with those of this study. We compared our patients’ return to sports with that of patients subjected to traditional surgical treatment. According to recent systematic review [35], 89.8% of patients returned to sport, with 71.6% returning to pre-injury levels. The rate of return to sport of our patients was higher, reaching 92.10% of the cohort, with 65.79% returning to the pre-injury levels. It is interesting to note that three (nearly 15%) patients were unable to return to the same level as before the operation and that the patients with lower functional scores had high TKS scores. This suggests that it is the psychological component (the fear of a new injury), rather than the functional inability to affect the score. Six months after isolated biological treatment, a cycle of three PRP injections [33], achieved a TAS score of 7.7 (SD 1.6), a satisfaction of 80%, and a rate of return to sports of 80% (median time, 2 months).

Contrary to earlier notions, tendons are actually characterized by a considerable metabolic activity, as demonstrated by clear activity-related circulatory responses and changes in collagen turnover. Indeed, in 2005, Alfredson [36] had already documented that chronic PT and Achilles tendinopathy are not associated with an inflammatory infiltrate or PGE2-mediated intra-tendinous inflammation, making corticosteroid treatment useless. Further tissue biology studies have highlighted a complex regulation of growth factors in normal tissue and in the reaction to tissue damage, showing an important role and effectiveness for growth factor application in the healing of injured tissue [37].

There are few studies of the application of PRFM systems to the patellar tendon. In a 2010 in vitro study, Visser et al. [37] analyzed the mitogenic power and concentration of PRFM growth factors in a canine patellar tendon model. The authors found that PRFM application resulted in a substantially increased number of released growth factors and in higher cell density compared to blood clot (BC). They concluded that these histological findings could be useful in the healing process of biologically compromised tissues. Another study tested a compact platelet-rich fibrin scaffold (CPFS) produced from blood and calcium gluconate in a rabbit model of a patellar tendon defect where the comparison was the untreated contralateral tendon [10]. The authors documented the potential of the CPFS as a provisional bioscaffold that was able to accelerate the repair of the injured patellar tendon, although the untreated tendon might eventually reach similar mechanical properties.

A larger number of studies have examined the application of PRFM in other body districts with varying results. Regarding the Achilles tendon, there are both pre-clinical works on animal models [38,39] and clinical studies [22] that agree in concluding that orthobiologic augmentation enhances and supports tendon healing, promotes accelerated regeneration, and could result in significant functional improvements in term of the efficiency of motion. The reviews regarding the rotator cuff seem to be more critical. Important randomized controlled studies [40,41,42] have found that a PRFM system did not significantly improve rotator cuff healing or vascularity, perioperative morbidity, hand muscle strength, structural integrity, or clinical rating scale scores. In 2020, Kurnaz and Balta [19] investigated the effect of a PRFM on the healing of vertical medial meniscal tears in a rabbit model. They did not detect any statistically significant increase in meniscal tear healing compared with the surgery group. Saltzman et al. [43] demonstrated that PRFM augmentation did not appear effective on gluteus medius tendon repair in terms of pain or clinical evidence of re-tears, even though they postulated a role for the orthobiologic membrane in improving subjective outcomes of overall and hip-specific physical functioning. In contrast, Abdelhakiem et al. [44] found that the addition of PRF after suturing the donkey superficial digital flexor tendon enhanced cell alignment and collagen deposition at the site of the injury, promoting tendon healing.

The interest in developing new technologies in the field of regenerative medicine is due to the promising outcomes of the earlier products, such as PRP, and the scope for further enhancing their clinical benefits.

According to a systematic review and meta-analysis by Barman et al. [45], PRP injections in PT do not seem to provide significantly greater clinical benefit in terms of pain relief and functional outcomes than does a lack of PRP treatment. However, when assessing PRP efficacy, it is fundamental to establish standardized PRP preparation and administration protocols in order to optimize its effectiveness. In 2024, Bosco et al. [46] conducted a comprehensive review of efficacy, protocols, and future directions regarding the use of PRP for jumper’s knee. Their study led to the conclusion that PRP demonstrates promising results in promoting cellular remodeling and accelerating the healing process in PT and shows potential in pain reduction, improved function, and accelerated recovery.

Based on these considerations, PRFM is a new generation platelet-based product that provides a three-dimensional fibrin matrix. It is macroscopically denser and more elastic than PRP preparations. The main advantage of PRFM over PRP is that the PRFM fibrin matrix entraps released thrombocytes and growth factors and then gradually supplies them to the wound site over time. Among the bioactive molecules stored and released by platelet α-granules, the ones that are commonly held to provide the greatest benefits for tissue regeneration are PDGF, IGF, TGF-β, VEGF, FGF, and ECGF [47]. Briefly, TGF-β increases the expression of procollagen types I and III and improves tendon mechanical properties as well as long-term healing [46,48,49]. PDGF, IGF-1, and VEGF promote angiogenesis and vasculogenesis, tendon cell proliferation, and tendon healing [50]. Chan et al. [51] showed in a rat patellar tendon model that basic FGF (bFGF) enhanced a dose-dependent increase in proliferating cells and type III collagen. Because of the relatively short half-life and rapid degradation of most growth factors, the local administration of PRP alone may not be able to provide a prolonged release of chemotactic and mitogenic factors, which in fact may be those particularly desired in biologically compromised tissues. Therefore, the fibrin scaffold serves first as a reservoir for cytokines, which are bound in the fibrin scaffold and are released gradually. As a result, the creation of a dense, platelet-rich, fibrin construct may provide a reservoir of growth factors that ensures the sustained elution of increased concentrations of growth factors over time [37].

Secondly, during the initial wound healing phase, platelets interact with the fibrin clot not only to provide a hemostatic plug, but also by creating a provisional fibrin scaffold that supports and stimulates cell migration and proliferation [52].

Thirdly, Andia et al. [53] have advanced the theory that PRP products can modify the biological status of injured tissue by occupying some of the physical space, thereby altering the cytokine profile and removing signals of inflammation or extracellular matrix catabolism. This “steric washout mechanism” is favored using high volumes and might be assimilated to saline injections (‘placebo’). In the case of PRFM, this mechanism is significantly enhanced by the structurally larger volume provided by the fibrin matrix to the platelet concentrate.

Finally, thanks to its three-dimensional morphology PFRM is easily sutured at the site chosen by the operator, thereby concentrating all its beneficial effects at the affected site.

A key advantage, shared by PRFM and PRP, is their autologous nature, which minimizes the risk of adverse reactions, such as pathogen transmission or immune responses. In addition, both are easy to prepare in an outpatient setting, making them a convenient and cost-effective treatment option [46]. Notably, both techniques are easily reproducible and minimally invasive, involving no donor-site morbidity or preoperative planning and requiring no surgical expertise.

The main limitation of our study stems from its retrospective nature, with the inherent biases in patient selection and data analysis. Moreover, a limited number of patients met our and the manufacturer’s inclusion and exclusion criteria. Lastly, we had no control group, whether involving other effective regenerative medicine therapies or solely surgical treatment.

In conclusion, this retrospective observational study, shows the safety and efficacy of surgical debridement and autologous PRFM application in patients with patellar tendinopathy, with a significant improvement in clinical outcomes as measured by the VAS, the VISA-P, the TAS, and Blazina scores at 24 months. These findings support the reliability and success of this biological treatment for patellar tendinopathy, with an excellent rate of return to sport, TKS score and patient satisfaction. Further studies are needed to validate these results and explore medium- and longer-term outcomes.

## Figures and Tables

**Table 1:** Preoperative and perioperative data.

Variable	Patients
Number	38
Age, mean (SD ^1^) [range]	31.32 (11.44) [17–50]
Gender	
Male (%)	28 (73.68)
Female (%)	10 (26.32)
Side	
Right (%)	20 (52.63)
Left (%)	18 (47.37)
BMI ^2^ (kg/m^2^), mean (SD) [range]	24.78 (1.99) [21.89–29.12]
Sport	
Volleyball (%)	15 (39.47)
Soccer (%)	15 (39.47)
Ciclismo (%)	2 (5.26)
Rugby (%)	2 (5.26)
Jogging (%)	2 (5.26)
Tennis (%)	1 (2.64)
Motocross (%)	1 (2.64)
ASA ^3^ class	
ASA 1 (%)	34 (89.47)
ASA 2 (%)	4 (10.53)
Operative time (min), mean (SD) [range]	39.68 (15.06) [20–70]

^1^ SD: standard deviation; ^2^ BMI: body mass index; ^3^ ASA: American Society of Anesthesiology.

**Table 2:** Postoperative functional tests and patient satisfaction before the procedure and at 24 months.

	Values	*p*-Value
VAS ^1^		<0.001
Baseline, mean (SD ^2^) [range]	8.58 (0.89) [7–10]	
24 months follow-up, mean (SD) [range]	1.68 (2.08) [0–5]	
VISA-P ^3^		<0.001
Baseline, mean (SD) [range]	58.05 (21.69) [15–82]	
24 months follow-up, mean (SD) [range]	80.32 (20.58) [35–100]	
TAS ^4^		<0.001
Baseline, mean (SD) [range]	7.79 (1.53) [4–10]	
24 months follow-up, mean (SD) [range]	7.08 (1.94) [4–10]	
Blazina score		<0.001
Baseline, mean (SD) [range]	3.16 (0.75) [2–4]	
24 months follow-up, mean (SD) [range]	0.68 (1.09) [0–3]	

^1^ VAS: Visual Analogue Scale; ^2^ SD: standard deviation; ^3^ VISA-P: Victorian Institute of Sport Assessment Scale for Patellar Tendinopathy; ^4^ TAS: Tegner Activity Scale.

**Table 3:** Return to sport at 24 months.

	Values
Return to sport	
No (%)	3 (7.90)
Yes (%)	35 (92.10)
Time of return to sport 24-month	87.83 (41.92) [60–240]
follow-up, mean (SD ^1^) [range]	
TKS ^2^	
24-month follow-up, mean (SD) [range]	19.53 (8.09) [13–37]
Satisfaction	
24-month follow-up, mean (SD) [range]	9.21 (1.21) [6–10]

^1^ SD: standard deviation; ^2^ TKS: Tampa Scale of Kinesiophobia.

## Data Availability

Data are available upon reasonable request.
